# Effective management of pembrolizumab-induced Stevens-Johnson syndrome/toxic epidermal necrolysis overlap with upadacitinib

**DOI:** 10.1016/j.jdcr.2025.11.011

**Published:** 2025-11-19

**Authors:** Lucas Goetz, Bo Wang

**Affiliations:** a University of South Dakota Sanford School of Medicine, Vermillion, South Dakota; b Avera Medical Group Dermatology, Aberdeen, South Dakota

**Keywords:** dermatologic toxicity, immune checkpoint inhibitor, Janus kinase inhibitor, pembrolizumab, SCORTEN, Stevens-Johnson syndrome, toxic epidermal necrolysis

## Introduction

Stevens-Johnson syndrome (SJS) and toxic epidermal necrolysis (TEN) are rare, life-threatening mucocutaneous reactions characterized by widespread epidermal necrosis, skin detachment, and mucosal involvement. Most cases are drug-induced and classified by body surface area involvement: SJS affects <10%, TEN >30%, and SJS/TEN overlap falls in between.^1^

Common triggers include antiepileptics, sulfonamides, and other antibiotics.^1^ Although immune checkpoint inhibitors (ICIs) such as pembrolizumab are widely used to treat malignancies, they are only rarely linked to SJS/TEN. A pooled safety analysis and pharmacovigilance data suggest increased risk with ICIs, though the incidence remains low.^2^

Given the overlap of features with other severe cutaneous reactions such as erythema multiforme major, histopathologic confirmation is essential.^1^

The pathogenesis involves drug-specific cytotoxic T cells inducing keratinocyte apoptosis via granulysin and Fas-Fas ligand pathways.^1^ Mortality correlates with disease severity, reaching up to 26% in TEN.^1^ Early recognition and treatment, withdrawal of the causative agent, and supportive care are central to management. In steroid-refractory cases, immunomodulatory agents, including intravenous immunoglobulin, tumor necrosis factor inhibitors, and Janus kinase (JAK) inhibitors, have been used with variable success.^3^

Here, we present a challenging case of SJS/TEN overlap induced by pembrolizumab and successfully treated with a combination of systemic steroids and upadacitinib. This case adds to the limited literature on ICI-induced SJS/TEN managed with a JAK inhibitor following steroid failure.

## Case presentation

A 51-year-old male with a history of squamous cell carcinoma of the left upper lobe of the lung presented with a progressive rash after initiating immunotherapy. He had completed concurrent chemotherapy with weekly paclitaxel and carboplatin, alongside radiation therapy. Other medications that had been started more than 1 month before presentation included famotidine, diphenhydramine, montelukast, albuterol, and dexamethasone as part of his chemotherapy regimen. As part of his adjuvant cancer treatment, he was started on pembrolizumab.

Approximately 5 days after initiating pembrolizumab, the patient developed pruritic and mildly painful erythematous plaques on his chest and upper back. He was evaluated at urgent care and prescribed prednisone 20 mg daily for 5 days. Over the next three days, his rash worsened, prompting oncology to refer him urgently to dermatology. He denied oral ulcers, eye pain, fever, and malaise. The initial workup showed no significant abnormalities in the metabolic panel or blood counts.

**Fig 1:**
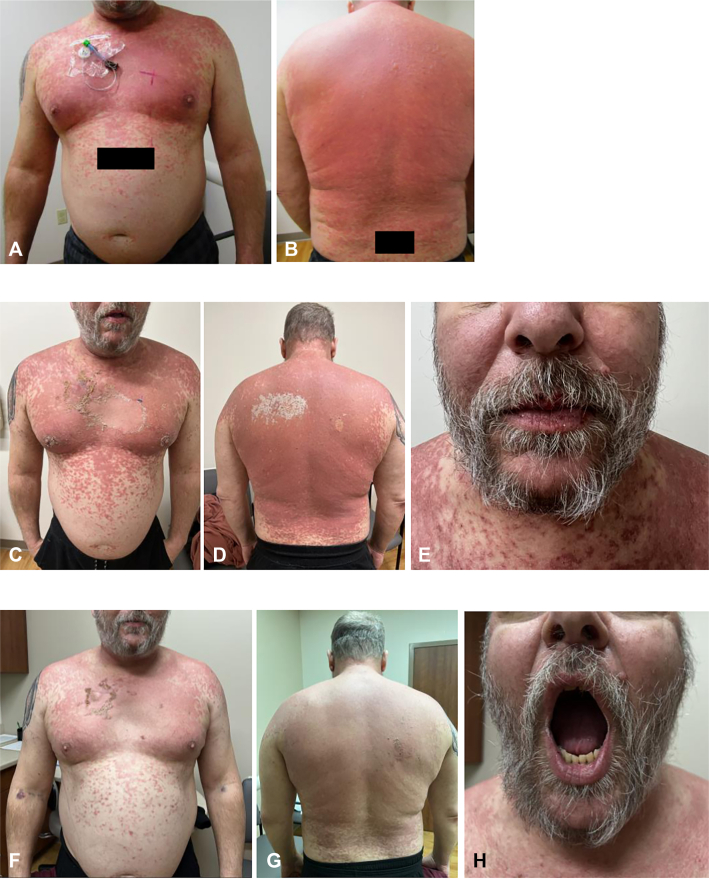
Rash progression over time in a patient with SJS/TEN overlap. **A** and **B,** Six days after rash onset. **C-E,** Eleven days after rash onset. **F-H,** Twenty days after rash onset. *SJS*, Stevens-Johnson syndrome; *TEN*, toxic epidermal necrolysis.

**Fig 2:**
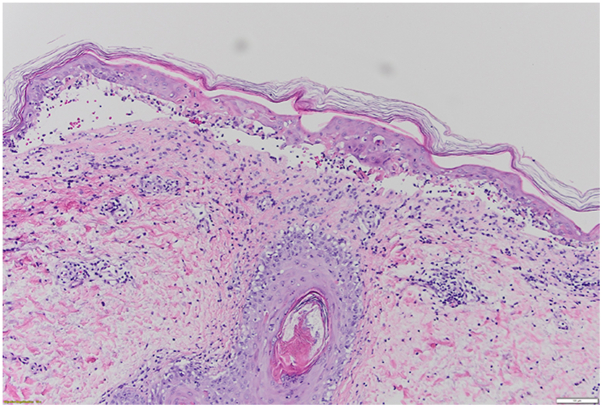
Hematoxylin and eosin (H&E) staining of skin biopsy revealed interface dermatitis with full-thickness epidermal necrosis, consistent with SJS/TEN overlap. *SJS*, Stevens-Johnson syndrome; *TEN*, toxic epidermal necrolysis.

On dermatologic exam, he had scattered erythematous and dusky papules coalescing to plaques over the upper trunk and proximal extremities (Fig 1, *A* and *B*), with early mucosal involvement of the lips but no intraoral lesions. The patient also reported mild ocular irritation without conjunctival injection or visual changes. He did not experience odynophagia or dysuria, and no additional mucosal findings developed following initiation of therapy. A 4-mm punch biopsy from the chest revealed interface dermatitis with full-thickness epidermal necrosis (Fig 2), consistent with SJS/TEN overlap. Clinical history and timing supported pembrolizumab as the likely offending agent.

Due to high clinical suspicion of SJS/TEN, the patient received IV methylprednisolone 125 mg daily for 2 days. Despite this, his rash progressed with further desquamation with a positive Nikolsky sign and new oral erosions (Fig 1, *C*-*E*). Given the extent and progression, he was admitted for intensive management. At that time, his SCORTEN score was 3, corresponding to an estimated 35% mortality risk.

Inpatient treatment included high-dose IV methylprednisolone 125 mg every 6 hours with supportive care and empiric ceftriaxone. Due to a limited response to steroids, upadacitinib 15 mg daily was initiated based on emerging evidence supporting its use in severe cutaneous adverse reactions.^4^ A 2-week sample course was provided and monitored by dermatology and pharmacy.

The patient tolerated the regimen well. His rash gradually improved, with re-epithelialization noted during hospitalization (Fig 1, *F*-*H*). He remained hemodynamically stable, with no new mucosal lesions or secondary infections. He was transitioned to oral prednisone (initially 80 mg daily) and discharged with a slow taper. The 2-week upadacitinib course was completed without adverse effects.

**Fig 3:**
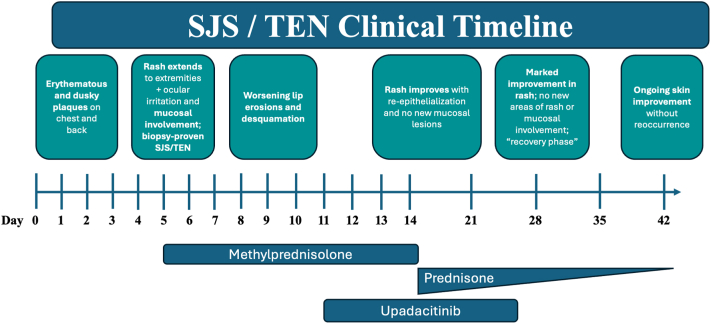
Clinical timeline showing rash progression and immunosuppressive treatments. *SJS*, Stevens-Johnson syndrome; *TEN*, toxic epidermal necrolysis.

At his 1-week dermatology follow-up, he reported marked improvement in skin symptoms and pruritus. No recurrent rash or mucosal involvement was noted. Approximately 6 weeks postdischarge, he continued to improve and remained under dermatologic and primary care follow-up, with no plans to resume pembrolizumab. A visual timeline of the clinical course is shown in Fig 3.

## Discussion

Pembrolizumab is a programmed death-1 (PD-1) immune checkpoint inhibitor widely used in advanced squamous cell carcinoma of the lung, often in combination with chemotherapy.^5^ Like other ICIs, pembrolizumab is associated with immune-related adverse events, with cutaneous reactions among the most frequent. By blocking PD-1 and enhancing T cell-mediated responses, pembrolizumab can disrupt immune tolerance and trigger hypersensitivity reactions such as SJS. Biopsies from patients with PD-1/PD-L1 inhibitor-induced SJS have shown increased CD8+ T cell infiltration at the dermal-epidermal junction.^6^

There is no standard treatment for SJS/TEN. Management typically includes early recognition, withdrawal of the offending agent, supportive care, and immunosuppression. High-dose corticosteroids are commonly used, though optimal dosing and duration remain debated due to potential adverse effects. Adjunctive therapies such as intravenous immunoglobulin and cyclosporine are also used.^6^ The SCORTEN score is a validated tool to estimate mortality risk in SJS/TEN. In this case, the patient’s score was 3 (patient’s age, extensive skin involvement, and underlying malignancy), corresponding to an estimated 35.3% mortality risk.^7^

Recent insights identify JAK-STAT signaling as a key mediator of keratinocyte death via interferon activation. Spatial proteomics in TEN lesions revealed strong upregulation of JAK-STAT-driven inflammation.^4^ JAK inhibitors, including tofacitinib, baricitinib, abrocitinib, and upadacitinib, block CD8+ T cell-mediated apoptosis. In this case, high-dose steroid monotherapy failed to suppress disease progression, prompting the addition of upadacitinib 15 mg daily, which successfully halted disease progression and induced re-epithelialization. Notably, disease progression was slower than usual, possibly due to prior dexamethasone use during chemotherapy and early prednisone (20 mg daily) prescribed at urgent care, which may have partially suppressed the immune cascade. Given the immunosuppressive nature of JAK inhibitors, prophylactic antibiotics, close monitoring, and a cautious taper are recommended. While early clinical experiences are promising, further clinical trials are needed to evaluate JAK inhibitors’ efficacy relative to traditional therapies and clarify their role in the acute management of SJS and TEN.^4^

## Conflicts of interest

None disclosed.
